# Two Novel Point Mutations in Clinical *Staphylococcus aureus* Reduce Linezolid Susceptibility and Switch on the Stringent Response to Promote Persistent Infection

**DOI:** 10.1371/journal.ppat.1000944

**Published:** 2010-06-10

**Authors:** Wei Gao, Kyra Chua, John K. Davies, Hayley J. Newton, Torsten Seemann, Paul F. Harrison, Natasha E. Holmes, Hyun-Woo Rhee, Jong-In Hong, Elizabeth L. Hartland, Timothy P. Stinear, Benjamin P. Howden

**Affiliations:** 1 Department of Microbiology, Monash University, Clayton, Victoria, Australia; 2 Infectious Diseases Department, Austin Health, Heidelberg, Victoria, Australia; 3 Microbiology Department, Austin Health, Heidelberg, Victoria, Australia; 4 Department of Microbiology and Immunology, University of Melbourne, Victoria, Australia; 5 Victorian Bioinformatics Consortium, Monash University, Clayton, Victoria, Australia; 6 Department of Chemistry, Seoul National University, Seoul, Korea; Dartmouth Medical School, United States of America

## Abstract

*Staphylococcus aureus* frequently invades the human bloodstream, leading to life threatening bacteremia and often secondary foci of infection. Failure of antibiotic therapy to eradicate infection is frequently described; in some cases associated with altered *S. aureus* antimicrobial resistance or the small colony variant (SCV) phenotype. Newer antimicrobials, such as linezolid, remain the last available therapy for some patients with multi-resistant *S. aureus* infections. Using comparative and functional genomics we investigated the molecular determinants of resistance and SCV formation in sequential *S. aureus* isolates from a patient who had a persistent and recurrent *S. aureus* infection, after failed therapy with multiple antimicrobials, including linezolid. Two point mutations in key staphylococcal genes dramatically affected clinical behaviour of the bacterium, altering virulence and antimicrobial resistance. Most strikingly, a single nucleotide substitution in *relA* (SACOL1689) reduced RelA hydrolase activity and caused accumulation of the intracellular signalling molecule guanosine 3′, 5′-bis(diphosphate) (ppGpp) and permanent activation of the stringent response, which has not previously been reported in *S. aureus*. Using the clinical isolate and a defined mutant with an identical *relA* mutation, we demonstrate for the first time the impact of an active stringent response in *S. aureus*, which was associated with reduced growth, and attenuated virulence in the *Galleria mellonella* model. In addition, a mutation in *rlmN* (SACOL1230), encoding a ribosomal methyltransferase that methylates 23S rRNA at position A2503, caused a reduction in linezolid susceptibility. These results reinforce the exquisite adaptability of *S. aureus* and show how subtle molecular changes cause major alterations in bacterial behaviour, as well as highlighting potential weaknesses of current antibiotic treatment regimens.

## Author Summary

The treatment of serious infections caused by *Staphylococcus aureus* is complicated by the development of antibiotic resistance, and in some cases the appearance of more persistent bacteria that have a reduced growth rate resulting in small colony variants (SCV). Here we have shown using whole genome sequencing and gene replacement experiments on sequential *S. aureus* isolates obtained from a patient with a serious bloodstream infection, how *S. aureus* evolved into a multi-antibiotic resistant, persistent and almost untreatable SCV. Specifically we show that a minor DNA change in a *S. aureus* gene encoding an enzyme called RelA causes an accumulation of a small signalling molecule called (p)ppGpp, which in turn leads to persistent activation of the important bacterial stress response known as the stringent response. This is the first report of the involvement of the stringent response in *S. aureus* SCV formation and its association with persistent infection. Additionally, we have uncovered a novel mechanism of resistance to the new antimicrobial linezolid, caused by a mutation in a gene encoding a 23S rRNA methyltransferase. This study highlights the exquisite adaptability of this important pathogen in the face of antimicrobial treatment.

## Introduction

The factors promoting persistence of bacterial infection in the face of apparently effective antimicrobial therapy have not been clearly defined. This particularly applies to *Staphylococcus aureus*, especially methicillin-resistant *S. aureus* (MRSA), which remains a major human pathogen that frequently causes invasive disease, often associated with a high mortality rate [1], [2], [3]. A number of bacterial factors have been associated with persistent bacteremia and failed antimicrobial therapy for serious MRSA infections, including reduced activity of the quorum sensing system *agr*, resistance to host antimicrobial peptides, and the evolution of reduced vancomycin susceptibility in patients treated with this antibiotic [4], [5]. Although traditionally considered an extracellular organism, recently it has been demonstrated that *S. aureus* can reside and persist in an intracellular state [6]. A staphylococcal phenotype that appears to be particularly associated with cellular invasion and clinical persistence is the small colony variant (SCV) phenotype [6], [7]. This is phenotypically characterised by reduced growth rate, small colony size and in some cases auxotrophism for hemin or menadione, related to mutations in genes encoding products involved in the electron transport system. Small colony variants of *S. aureus* have been associated with persistent and recurrent *S. aureus* infections, and with increased antimicrobial resistance [7]. The mechanisms of the SCV phenotype in *S. aureus* have been investigated in detail over a number of years. Defined *hemB* and *menD* mutants [6], [7] of laboratory *S. aureus* strains have defects in electron transport, and have demonstrated global transcriptional changes [8], increased cellular attachment, invasion and persistence [9], [10], [11], reduced antibiotic susceptibility [12], and reduced virulence [13]. However, despite this significant work the molecular correlates of persistence have not been definitively elucidated in clinical isolates of *S. aureus*.

One important bacterial response to stress and nutritional starvation, including antimicrobial challenge, is activation of the stringent response, mediated by intracellular accumulation of the alarmones ppGpp and pppGpp [(p)ppGpp], which are usually controlled by the activities of a synthetase (RelA) and a hydrolase (SpoT) [14]. In gram positive organisms, including *S. aureus*, a single *rel* gene encodes a protein with synthetase and hydrolase domains that controls the stringent response under stressful conditions, while other synthetases such as RelP and RelQ provide basal levels of (p)ppGpp during non stressful conditions [14], [15], [16], [17]. The stringent response has been associated with persistence of infection in *Mycobacterium tuberculosis*, where it is important for the long term survival of the organism [18], and recently has been linked with growth defects and vancomycin tolerance in *E. faecalis* [17]. However, different bacteria have developed different strategies to utilize the alarmones in intracellular signalling, with diverse regulatory changes found in different organisms [19]. The impact of an active stringent response has not been studied in *S. aureus*, mainly because a functional hydrolase domain of the RelA/SpoT homolgue in *S. aureus* (RelA) is essential for survival of the organism [19], [20], [21]. Although mupirocin is a strong inducer of the stringent response in *S. aureus* and has been used to investigate the transcriptional profile of an active stringent response in this organism, it also leads to RelA/SpoT independent transcriptional changes [19], [22], indicating that the mupirocin model alone is not an optimal strategy to study the stringent response in this organism. Although it could be anticipated that the bacterial stringent response would play a role in the adaptation of *S. aureus* to antimicrobial challenge during persistent infection, this has not been previously reported.

For many years vancomycin has been the mainstay of therapy for serious MRSA infections [5]. However, with increasing antimicrobial resistance in MRSA, including resistance to vancomycin, the newer, novel antimicrobials such as linezolid and daptomycin are the last available therapies in some patients [5]. While there are increasing reports of daptomycin non-susceptible *S. aureus* strains [23], reports of reduced linezolid susceptibility in *S. aureus* have, to date, been rare. Linezolid is the first in a new class of antimicrobials, the oxazolidinones, that bind to the A site of the peptidyl transferase centre (PTC) of the bacterial ribosome [24], inhibiting bacterial ribosomal protein synthesis. Resistance to linezolid in *S. aureus* has primarily been related to target site mutations in domain V of 23S rRNA, especially the G2576U mutation [25], [26]. Recently, however, a naturally occurring resistance gene *cfr*, which encodes Cfr methyltransferase and leads to modification of adenosine at position 2503 in 23S rRNA has been described in a single *S. aureus* isolate from Columbia, and in two staphylococcal clinical isolates from the USA [27], [28]. The *cfr* gene on the chromosome was associated with mobile genetic elements, suggesting the resistance mechanism may be transferable [27]. The *S. aureus* genome encodes a number of conserved RNA methyltransferases, including RlmN (encoded by SACOL1230), and although methylation of rRNA is a common mechanism of acquired antimicrobial resistance [29], mutations in chromosomally encoded RNA methyltransferases have not been linked to reduced linezolid susceptibility in *S. aureus*.

Recently, we treated a patient with persistent and recurrent methicillin-resistant *S. aureus* (MRSA) bacteremia despite extensive, appropriate antimicrobial therapy. The clinical isolates obtained following treatment demonstrated significant antimicrobial resistance, including reduced susceptibility to linezolid, and features characteristic for small colony variant strains (SCV) of *S. aureus* [6], [7]. However, phenotypic features suggested that mutations were not present in hemin or menadione biosynthesis genes. Therefore we investigated the mechanisms of persistence and antimicrobial resistance in these isolates using a combined comparative and functional genomics approach, and discovered a clinical isolate with a persistently activated stringent response, and a novel mechanism of reduced linezolid susceptibility.

## Results

### Clinical details

**Figure 1:**
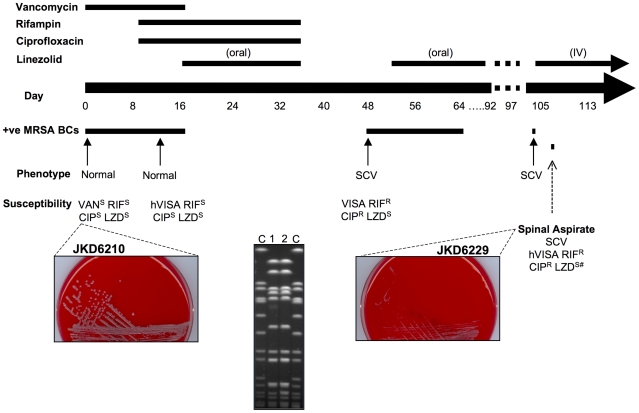
Clinical features and antimicrobial therapy. The major features of the clinical case and the relevant clinical isolates are demonstrated. Included are photos of overnight cultures on HBA depicting the normal MRSA strain (JKD6210) and the SCV strain (JKD6229) isolated after many weeks of failed antimicrobial therapy. Pulsed field gel electrophoresis results of *Sma*I digested DNA from the paired clinical isolates are also included (lane 1 JKD6210, lane 2 JKD6229) and demonstrate an identical banding pattern for the two strains. #Note, JKD6229 demonstrated reduced linezolid susceptibility within the susceptible MIC range.

A 73-year old man with end-stage renal failure was admitted with line related methicillin-resistant *S. aureus* (MRSA) bacteremia. The MRSA was susceptible to clindamycin, trimethoprim-sulfamethoxazole, ciprofloxacin, vancomycin, rifampicin and fusidic acid. He was commenced on intravenous vancomycin, and due to persistent *S. aureus* bacteremia, rifampin and ciprofloxacin were added. After 16 days of ongoing bacteremia and detection of heterogeneous vancomycin-intermediate *S. aureus* (hVISA), vancomycin was changed to oral linezolid and he completed 18 days of linezolid combined with rifampicin and ciprofloxacin. Multiple investigations including transesophageal echocardiogram, computed tomography of brain, chest, abdomen, pelvis and lumbar spine, and white cell/SPECT imaging did not reveal any definite focus. Eleven days later he developed fever, hypotension and back pain and blood cultures were again positive for MRSA, on this occasion a small colony variant (SCV). He was recommenced on oral linezolid and completed 6 weeks of therapy. Five days later he developed severe lumbar back pain and raised inflammatory markers. A single blood culture and a lumbar aspirate from the L3–4 region again cultured SCV-MRSA (Fig. 1). He was commenced on intravenous linezolid and completed 6 weeks of therapy, and was changed to trimethoprim-sulfamethoxazole for long-term suppressive treatment. Pulsed field gel electrophoresis demonstrated that the SCV strain JKD6229 emerged from the parental strain (JKD6210) [5] (Fig. 1), but JKD6229 did not demonstrate auxotrophism for hemin or menadione [7]. Both strains were multi-locus sequence type 5. During failed therapy, resistance to ciprofloxacin, rifampin and reduced susceptibility to linezolid developed (Table 1).

**Table 1:** Strains and plasmids used in this study.

		MIC (**µ**g/mL)								
Strain or plasmid	Properties	VAN^a^	TEI^a^	GEN	LIN^a^	DAP	TIG	RIF	CIP	Ref
**Strain**										
** Isolate Pair**										
JKD6210	VSSA, RIF^S^, CIP^S^. Day 0 BC isolate	4.0	3.0	0.38	0.75	0.19	0.19	0.023	0.5	This study
JKD6229	SCV, RIF^R^, CIP^R^. Day 107 spinal aspirate	8.0	8.0	0.38	2.0	0.38	0.19	>256	2.0	This study
**Others**										
JKD6301	JKD6210 with point mutation in *relA* from JKD6229	4.0	3.0	0.50	0.75	0.19	0.19	0.023	0.5	This study
JKD6300	JKD6210 with “CAA” insertion in SACOL1230 from JKD6229	NT	NT	NT	2.0	NT	NT	NT	NT	This study
RN4220	*S. aureus* strain capable of stably maintaining shuttle plasmids									[71]
P1	Capsule type 8 positive									[72]
Newman	Capsule type 5 positive									[72]
*E. coli* DH5α										NEB
**Plasmids**										
pKOR1	*E. coli*/*S. aureus* shuttle vector for the construction of allelic-exchange mutants									[39]
pJKD6318	pKOR1 with the *relA* loci from JKD6229, generated with oligos P-relA-F-AttB1 and P-relA-R-AttB2									This study
pJKD6319	pKOR1 with the SACOL1230 loci from JKD6229, generated with oligos P-CAA-F-attB1 and P-CAA-R-AttB2									This study

NB. ^a^Vancomycin, Teicoplanin and Linezolid Etest performed with 2McF suspension. SCV, small colony variant. VAN, vancomycin; TEI, teicoplanin; GEN, gentamicin; LIN, linezolid; DAP, daptomycin; TIG, tigecycline; RIF, rifampin; CIP, ciprofloxacin; NT, not tested. NOTE, JKD6210 was vancomycin-susceptible by standard Etest and vancomycin population analysis profile (PAP) testing.

### Microarray transcriptional analysis

**Figure 2:**
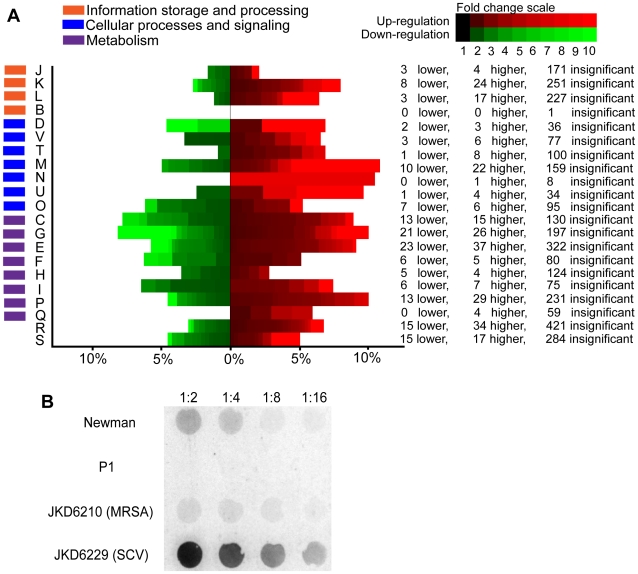
Microarray transcriptional analysis of SCV strain JKD6229 and parental strain JKD6210. **A**) Results of microarray transcriptional analysis of JKD6229 (SCV) compared to JKD6210 (MRSA). Up-regulated genes (in red) are differentially up-regulated in JKD6229 compared to the parent strain JKD6210, and the down-regulated genes (in green) are differentially down-regulated in JKD6229 compared to JKD6210. The heat map analysis highlights the proportion of each cluster of orthologous groups (COG) functional group [70] that is differentially regulated in the array analysis. This clearly demonstrates global transcriptional changes in the SCV strain, affecting genes from all COG groups. J is associated with translation, ribosomal structure and biogenesis; K is related to transcription; L is related to replication, recombination and repair; B is related to chromatin structure and dynamics; D is related to cell cycle control, cell division, chromosome partitioning; V is related to defence mechanisms, T is related to signal transduction mechanism; M is related to cell wall, membrane and envelope biogenesis; N is related to cell motility; U is related to intracellular trafficking, secretion, and vesicular transport; O is related to posttranslational modification, protein turnover, chaperones; C is related to energy production and conversion; G is related to carbohydrate transport and metabolism; E is related to amino acid transport and metabolism; F is related to nucleotide transport and metabolism; H is related to coenzyme transport and metabolism; I is related to lipid transport and metabolism; P is related to inorganic ion transport and metabolism; Q is related to secondary metabolites biosynthesis, transport and catabolism; R and S are function unknown or general function prediction only categories. **B**) Anti-capsule type 5 immunoblot of serial dilutions of crude capsule extracts from JKD6210, JKD6229, and control strains Newman (Cap5 positive) and P1 (Cap8 positive), demonstrating a marked increase in capsule production in the SCV strain JKD6229, consistent with the microarray transcriptional profiles.

Initially, to understand the molecular determinants of clinical persistence in the SCV-MRSA isolate JKD6229 the transcriptional profile was analysed. Using microarray transcriptional analysis significant global gene expression changes were found in the SCV strain (JKD6229) compared to the parental strain (JKD6210) (349 genes up-regulated and 175 genes down-regulated ≥2-fold (see Fig. 2, Table 2 and Table S1). Changes included pronounced up-regulation (up to 80-fold) of genes encoding capsule biosynthesis in JKD6229 (*cap5A* to *cap5P*; SAV0149 to SAV0164). To confirm the biological impact of capsule gene transcriptional changes the capsule type of JKD6210 and JKD6229 was confirmed as type 5 by PCR [30], and a capsule immunoblot was then performed. This demonstrated significant enhancement of capsule production in JKD6229 compared to the parental strain JKD6210 and the capsule type 5 control strain Newman (Fig. 2B).

**Table 2:** Selected genes differentially regulated in JKD6229 compared to JKD6210 based on microarray transcriptional analysis.

Locus_tag	Gene	Putative product	Fold ratio (JKD6229∶JKD6210)
**CELL ENVELOPE: ** ***Capsule Biosynthesis***			
SAV0149	*capA*	capsular polysaccharide synthesis enzyme Cap5A	13.3
SAV0150	*capB*	capsular polysaccharide synthesis enzyme Cap5B	10.9
SAV0151	*capC*	capsular polysaccharide synthesis enzyme Cap8C	12.0
SAV0152	*capD*	capsular polysaccharide synthesis enzyme Cap5D	60.2
SAV0153	*capE*	capsular polysaccharide synthesis enzyme Cap8E	25.7
SAV0154	*capD*	capsular polysaccharide synthesis enzyme Cap5F	80.7
SAV0155	*capF*	capsular polysaccharide synthesis enzyme Cap5G	23.0
SAV0156	*capG*	capsular polysaccharide synthesis enzyme Cap5H	19.0
SAV0157	*capI*	capsular polysaccharide synthesis enzyme Cap5I	39.3
SAV0158	*capJ*	capsular polysaccharide synthesis enzyme Cap5J	14.5
SAV0159	*capK*	capsular polysaccharide synthesis enzyme Cap5K	10.3
SAV0160	*capL*	capsular polysaccharide synthesis enzyme Cap5L	7.2
SAV0161	*capM*	capsular polysaccharide synthesis enzyme Cap5M	4.6
SAV0162	*capN*	capsular polysaccharide synthesis enzyme Cap5N	11.9
SAV0163	*capO*	capsular polysaccharide synthesis enzyme Cap5O	2.9
**REGULATORY FUNCTIONS:**			
SAS1940a	*hld*	delta-hemolysin precursor	10.3
SAV2036	*agrB*	accessory gene regulator B	3.0
SAV2037	*agrD*	accessory gene regulator D	2.2
SAV2038	*agrC*	accessory gene regulator C	6.4
SAV2039	*agrA*	accessory gene regulator A	2.8
**CELLULAR PROCESSES: ** ***Pathogenesis***			
SAV0111	*spa*	Immunoglobulin G binding protein A precursor	0.4
SAV2502	*fnbB*	fibronectin-binding protein homolog	2.4
SAV2503	*fnbA*	fibronectin-binding protein homolog	2.8
***Toxin production***			
SA1761	*sep*	enterotoxin P	2.8
SAV1163	*hly*	alpha-hemolysin precursor	6.0
**Superantigen-like**			
SAS0387		Exotoxin	0.5
SAV0433	*set15*	Exotoxin 15	0.3
**CARBOHYDRATE TRANSPORT AND METABOLISM:**			
**Down-regulated**			
SACOL2180	*lacG*	6-phospho-beta-galactosidase	0.03
SAR2281	*lacE*	PTS system, lactose-specific IIBC component	0.4
SAS0164		glucose-specific PTS transporter protein, IIABC component	0.2
SAS2090		6-phospho-beta-galactosidase	0.04
SAS2096		galactose-6-phosphate isomerase	0.01
SAV0189	*glcA*	glucose-specific PTS enzyme II	0.2
SAV0242		maltose and glucose-specific PTS enzyme II	0.1
SAV0962	*pgi*	glucose-6-phosphate isomerase	0.3
SAV2189	*lacG*	6-phospho-beta-galactosidase	0.01
SAV2190	*lace*	PTS system, lactose-specific IIBC component	0.02
SAV2191	*lacF*	PTS system, lactose-specific IIA component	0.02
SAV2192	*lacD*	tagatose 1,6-diphosphate aldolase	0.01
SAV2193	*lacC*	tagatose-6-phosphate kinase	0.01
SAV2194	*lacB*	galactose-6-phosphate isomerase	0.03
SAV2256		glucose uptake protein homolog	0.5
SAV2538	*ptsG*	PTS system, glucose-specific II ABC component	0.3
**Up-regulated**			
MW2435		fructose-bisphosphatase (fbp)	2.2
SAS0431		sugar-specific PTS transport system, IIBC component (trehalose)	2.2
SAS1448		maltose operon transcriptional repressor	7.5
SAV0247	*gatC*	probable PTS galactitol-specific enzyme IIC component	2.0
SAV0700	*fruA*	PTS system, fructose-specific IIBC component	2.2
SAV1507	*malA*	alpha-D-1,4-glucosidase	5.7
SAV1508	*malR*	maltose operon transcriptional repressor	8.0
SAV2377	*scrA*	PTS system, sucrose-specific IIBC component	3.0
SAV2506	*gntK*	gluconokinase	8.5
SAV2507	*gntR*	gluconate operon transcriptional repressor	4.1
SAV2516	*fbp*	fructose-bisphosphatase	3.6
**ENERGY METABOLISM: ** ***TCA Cycle***			
SAV1147	*sdhC*	succinate dehydrogenase cytochrome b-558	9.4
SAV1148	*sdhA*	succinate dehydrogenase flavoprotein subunit	2.9
SAV1413	*kgd*	alpha-ketoglutarate decarboxylase	2.1
SAV1695	*citZ*	methylcitrate synthase	5.8
SAV1791	*pckA*	phosphoenolpyruvate carboxykinase	2.3
**AMINO ACID METABOLISM:**			
SAV0962	*rocD*	ornithine—oxo-acid transaminase	2.64
SAV0986	*opp3B*	oligopeptide transport permease, Opp3B	2.53
SAV0987	*opp3C*	oligopeptide transport permease, Opp3C	4.94
SAV0988	*opp3D*	oligopeptide transport ATP-binding protein, Opp3D	7.27
SAV0989	*opp3F*	oligopeptide transport ATP-binding protein, Opp3F	7.36
SAV0990	*opp3A*	oligopeptide binding protein, Opp3A	6.06
SAV0994		oligopeptide transport system permease	3.19
SAV1023	*htrA*	Serine protease HtrA	3.34
SAV1083	*argJ*	bifunctional ornithine acetyltransferase/N-acetylglutamate synthase, ArgJ	2.61
SAV1085		ornithine aminotransferase	2.69
SAV2053	*ilvD*	dihydroxy-acid dehydratase	6.03
SAV2054	*ilvB*	acetolactate synthase large subunit	4.54
SAV2057	*leuA*	2-isopropylmalate synthase	7.07
SAV2058	*leuB*	3-isopropylmalate dehydrogenase	2.58
SAV2059	*leuC*	isopropylmalate isomerase large subunit	4.81
SAV2060	*leuD*	3-isopropylmalate dehydratase small subunit	3.32
SAV2062		hypothetical protein	2.01

NOTE, the full list of differentially regulated genes is available in Table S1.

Intracellular persistence and the SCV phenotype of *S. aureus* has previously been associated with down regulation or complete loss of activity of the global quorum sensing accessory gene regulator (*agr*) [31], however all genes encoding the *agr* locus (SAV2036 to SAV2039) and the delta-hemolysin precursor (SAS1940a) were significantly up-regulated in the SCV strain JKD6229 (2 to 10-fold increased expression). Associated with this was up-regulation of two genes encoding exotoxins (alpha-hemolysin [SAV1163], 5.9-fold increase; enterotoxin P [SA1761], 2.8-fold increase), however the SAV1163 orthologue in JKD6210 and JKD6229 was found to be a pseudogene because of a point mutation introducing a premature stop codon.

Distinct differential regulation of genes involved in carbohydrate transport and metabolism, amino acid metabolism and oligopeptide transport was also detected. Genes involved in lactose utilization and galactose metabolism (SAV2189-SAV2194) were remarkably down-regulated (up to 100-fold), with similar changes found in genes encoding key glycolysis enzymes such as *pgi* (SAV0962, glucose-6-phophate isomerase), while genes with products potentially involved in metabolism of alternative carbon sources such as sucrose, fructose and galactitol (*scrA, gatC, fruA, fruB*) were up-regulated in SCV JKD6229. Amino acid metabolism was another distinct functional class that was up-regulated in SCV JKD6229. Genes encoding valine, leucine and isoleucine biosynthesis enzymes showed increased expression, as did genes such as *rocD* (SAV0957) and *argJ* (SAV0183) linked to ornithine and arginine production. Striking too was the up-regulation of the Opp3 oligopeptide transport system (SAV0986–SAV0994). Opp3 facilitates the acquisition 4–8 aa-long peptides from the extracellular environment and it is the only known functional oligopeptide transport system in *S. aureus* [32].

### Whole genome comparison of SCV JKD6229 and parent strain JKD6210

**Figure 3:**
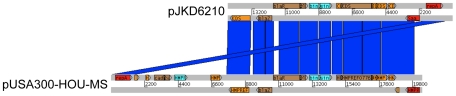
Sequence comparison of pJKD6210 and pUSA300-HOU-MS. Linear comparison (Artemis Comparison Tool) of the ∼15 kb plasmid detected in *S. aureus* JKD6210 (pJKD6210) compared with pUSA300-HOU-MS [33]. Replication and regulatory genes are colored red, recombination/transposition genes are blue, antibiotic/heavy metal/bacteriocin resistance genes are brown, hypothetical genes are colored orange. Blue vertical bars indicate the regions of pJKD6210 and pUSA300-HOU-MS sharing high DNA sequence identity. Note; *blaZ*, beta-lactamase; *blaR*, beta-lactamase regulator; bin, invertase; sin, recombinase; *repA*, replication protein.

The prominent transcriptional changes detected in JKD6229 compared to JKD6210 suggested SCV JKD6229 had undergone important genetic changes. In addition, the transcriptional profile of SCV JKD6229 was significantly different to the transcriptional profile of the SCV *hemB* mutant, suggesting that mutations in other genes may be contributing to the SCV phenotype of this strain [8]. Therefore whole genome sequencing and comparison of the parental MRSA strain JKD6210 and the SCV JKD6229 was performed. Illumina short-read sequencing yielded 2.7 Mb of mappable data for each genome. After detailed reciprocal sequence comparisons and comparisons against the reference genomes *S. aureus* COL and *S. aureus* N315, the only changes detected in JKD6229 compared to JKD6210 were two nucleotide substitutions, two codon insertions, and the loss of a ∼15 kb plasmid (Table 3). The sequences for JKD6210 and JKD6229 were aligned to the genome sequence of N315 (also MLST 5–the same as JKD6210 and JKD6229) and this demonstrated that 97.2% of N315 was covered to a depth of ≥20 in both JKD6210 and JKD6229. The regions not covered in N315 by the JKD6210 sequence were the same as the regions not covered by the JKD6229 sequence. PCR and Sanger sequencing confirmed the presence of each mutation in SCV JKD6229, and PCR and plasmid analysis confirmed the loss of the plasmid in JKD6229. Annotation and BLAST analysis of the plasmid (denoted as pJKD6210) revealed a pUSA300-HOU-MS-like replicon (Fig. 3) with genes encoding beta-lactam resistance, but absence of the genes encoding cadmium resistance that are present on pUSA300-HOU-MS [33]. All four changes in nucleotide sequence were associated with a predicted amino acid change or addition, suggesting one or more of these mutations might be responsible for the phenotypic changes in JKD6229.

**Table 3:** Summary of base substitutions and insertions detected in JKD6229 (small colony variant) compared to JKD6210 (methicillin-resistant *S. aureus*).

ORF ID	Gene	Gene Product	Mutation Type	Allele JKD6229 (no. reads)	Allele JKD6210 (no. reads)	Effect of Mutation
SACOL0588	*rpoB*	DNA-directed RNA polymerase	Substitution	T (302)	C (387)	His 481 Tyr
SACOL1230	*rlmN*	Ribosomal RNA large subunit methyltransferase	Insertion	CAA (60) “-“ (50)	“-“ (151)	Glu insertion prior codon 354
SACOL1390	*parC*	DNA topoisomerase IV subunit A	Insertion	TGT (93) “-“ (50)	“-“ (229)	Val insertion prior to codon 463
SACOL1689	*relA*	Guanosine polyphosphate pyrophosphohydrolase/synthetase	Substitution	T (176)	A (223)	Phe 128 Tyr

**NOTE:** A ∼15 kb plasmid was also deleted in JKD6229 (see text); “-“ means no insertion was present in those reads.

Two of the four mutations clearly corresponded with the acquired antibiotic resistance of SCV JKD6229. The change in *rpoB* that led to a H481Y substitution is a mutation commonly linked with rifampin resistance in *S. aureus* [34] and the amino acid insertion in *parC* (encoding Topoisomerase IV) likely contributed to reduced ciprofloxacin susceptibility in this strain. Single mutations in topoisomerase IV without additional mutations in DNA gyrase are often associated with low-level quinolone resistance [35], as demonstrated in JKD6229 (Table 1).

### A novel mutation in SACOL1230 contributed to reduced linezolid susceptibility in JKD6229

The ‘CAA’ insertion in SACOL1230, encoding RlmN, a ribosomal RNA large subunit methyltransferase, was associated with the linezolid exposure of SCV JKD6229. RlmN methylates 23S ribosomal RNA at adenosine 2503, and deletion of the gene renders *S. aureus* more susceptible to linezolid [36]. Linezolid resistance in clinical isolates of staphylococci is often linked to G2576T mutations in domain V of the 23S rRNA genes [37] or acquisition of a plasmid-encoded *cfr* (methyltransferase), which also methylates ribosomal RNA at position 2503 [28]. Interestingly, a 23S rRNA T2500A mutation has also been previously linked to linezolid resistance in a clinical isolate of *S. aureus* [38], but mutations in SACOL1230 have never been reported. The ‘CAA’ insertion in JKD6229 is predicted to incorporate an additional glutamate to the motif (DIDACCGQ’Q’) at the extreme C-terminus of the enzyme, a motif that is absolutely conserved among diverse Gram positive and negative bacteria [36]. An allelic replacement experiment was performed, where the normal SACOL1230 sequence from JKD6210 was replaced with the mutated SACOL1230 allele from JKD6229 using pKOR1 [39]. The SCV clinical strain (JKD6229) and the mutant JKD6300 (JKD6210 with SACOL1230 ‘CAA’ insertion) both demonstrated an increase in linezolid MIC within the susceptible range when an Etest using a 2 McFarland inoculum was used (Table 1).

### A mutation in SACOL1689 (*relA*) permanently activated the stringent response in SCV strain JKD6229

The fourth mutation occurred in SACOL1689. Based on high amino acid sequence similarity (71% amino acid similarity) to an ortholog in *Streptococcus mutans*, SACOL1689 (*relA*) is predicted to encode a bifunctional enzyme that modulates the amount of the intracellular signalling molecules guanosine 3′-diphosphate 5′-triphosphate and guanosine 3′, 5′-bis(diphosphate), abbreviated to (p)ppGpp [40]. Accumulation of (p)ppGpp, activates the bacterial stringent response leading to a switch to “survival mode” [17], [18]. The mutation in *relA* (SACOL1689) was of particular interest with respect to SCV formation because of the involvement of this gene in the bacterial stringent response and the potential impact on growth characteristics of an enhanced stringent response. Additionally, the microarray transcriptional profile of JKD6229 suggested the stringent response was active in this strain, with upregulation of amino acid catabolism pathways, significant over expression of genes encoding oligopeptide transport proteins, up-regulation of genes associated with isoleucyl tRNA limitation (including *ilvB* and *ilvD*, 4 to 6-fold increase; and *leuABCD*, up to 7-fold increase) [41], over expression of genes encoding extracellular proteases (*hrtA*, *splB*, SAV1612, SAV1613), and up-regulation of the quorum sensing system *agr*. The transcriptional profile was very similar to the profile of *S. aureus* after *in vitro* induction of the stringent response by exposure to mupirocin, an agent that inhibits isoleucyl tRNA synthetase [22].

**Figure 4:**
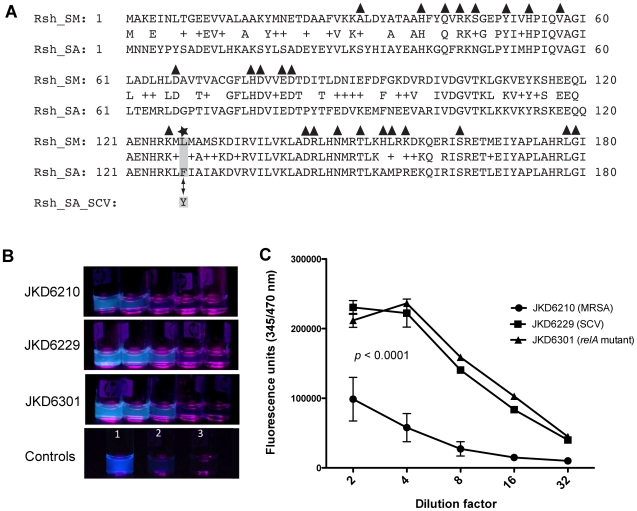
Location of RelA mutation in JKD6229 and impact of the mutation on cellular ppGpp levels. **A**) Alignment of the N-terminal Rsh domains of RelA/SpoT from *S. mutans* (Rsh_SM) and RelA from *S. aureus* JKD6210 (Rsh_SA). The triangles are regions shown by Hogg *et al*. [40] that-when mutated-affect hydrolase function. Indicated by star and grey shading is the F128Y amino acid substitution that occurs in SCV JKD6229 (Rsh_SA_SCV). **B and C**) Analysis of ppGpp levels in JKD6210 (MRSA), JKD6229 (SCV) and JKD6301 (JKD6210 with *relA* F128Y mutation) using the fluorescent chemosensor PyDPA [42]. **B**) Five, two-fold serial dilutions (1/2–1/32) of test strains demonstrate increased ppGpp levels by increased fluorescence in JKD6229 and JKD6301 compared with the parental strain JKD6210. Control 1 is JKD6210 exposed to serine hydroxymate; control 2 is JKD6210 without the addition of PyDPA; control 3 is buffer alone with PyDPA added. (C) Results confirmed in a 96-well plate format, analysed with a fluorescent plate reader. Results are presented as the mean±SD of biological replicates with a significant increase in fluorescence found for JKD6229 and JKD6301 compared to the parental strain JKD6210 (P<0.0001).

Structural and functional studies of RelA in *Streptococcus mutans* (RelA*_Sm_*) have shown that the enzyme can modulate intracellular levels of (p)ppGpp through a N-terminal hydrolase domain and C-terminal synthetase domain that act antagonistically in a ligand-dependant manner, either by degrading (p)ppGpp within the hydrolytic domain or converting GDP or GTP to (p)ppGpp within the synthetic domain [40]. Scanning mutagenesis of RelA*_Sm_* has defined regions of the enzyme that are critical for its hydrolytic function [40]. An alignment of the N-terminus of RelA from SCV JKD6229 (RelA_SCV_) with RelA*_Sm_* shows that the F128Y mutation occurred in a region known to be critical for hydrolase function in *S. mutans* (Fig. 4A). Thus, the alignment data and microarray results suggested that RelA_SCV_ might have impaired (p)ppGpp hydrolase function leading to an accumulation of (p)ppGpp and the persistent activation of the stringent response. To confirm that the RelA F128Y mutation was causing accumulation of (p)ppGpp, the *relA* allele from SCV JKD6229 was introduced into the parental strain JKD6210, and ppGpp levels were measured using the fluorescent chemosensor PyDPA [42]. As predicted, a significant increase in ppGpp levels was demonstrated in JKD6229 (clinical SCV) and the mutant RelA F128Y mutant JKD6301, compared to the parental strain JKD6210 (Fig. 4B and C), suggesting that the F128Y mutation reduced the hydrolase activity of RelA. This is the first time an activated stringent response has been implicated as a mechanism of SCV formation in clinical *S. aureus*.

### Phenotypic characteristics and virulence of *S. aureus* strains with a persistently activated stringent response

**Figure 5:**
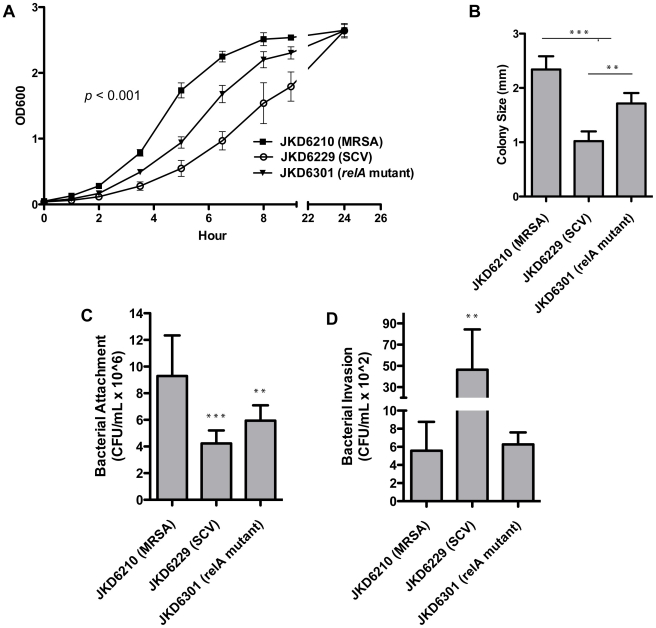
Phenotypic features and cellular attachment, invasion and persistence of clinical isolates and mutant strain JKD6301. **A and B**) Growth characteristics of parental strain JKD6210 (normal MRSA), the clinical SCV strain (JKD6229), and the allelic exchange *relA* mutant containing the F128Y mutation (JKD6301). Reduced growth rate in MH broth (**A**) and reduced colony size on HBA agar (**B**) is demonstrated for the SCV strain JKD6229. The *relA* mutant JKD6310 demonstrates reduced growth rate in broth and on solid media, but is not as impaired as the clinical SCV strain. The growth curves were significantly different for all strains (*P*<0.001). An analysis of cellular attachment/invasion after 1 hour incubation demonstrates a reduced rate of attachment in JKD6229 and JKD6301 compared to JKD6210 (**C**), however an analysis of cellular invasion after 2 hours incubation (**D**), demonstrates significantly greater invasion for the SCV strain JKD6229 compared to JKD6210 and JKD6301. Results are presented as mean±SD of triplicates from at least three independent experiments. **, *P*≤0.01; ***, *P*≤0.001.

**Figure 6:**
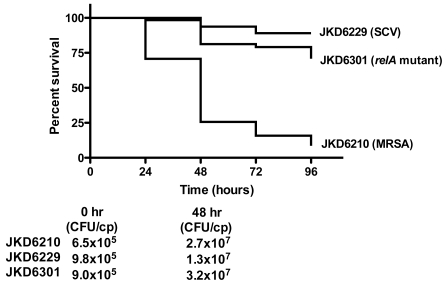
*Galleria mellonella* virulence assay. The percent survival of caterpillars injected with the clinical isolate pair (JKD6210, normal MRSA; JKD6229, SCV) and the *relA* mutant JKD6301 over 96 hours post injection is demonstrated using a Kaplan Meier plot. The average initial inoculum per caterpillar and the colony counts from selected worms after 48 hours incubation are also demonstrated. The difference in survival between JKD6210 and JKD6229 or JKD6301 was significant (*P*<0.0001), and the difference between JKD6229 and JKD6301 was also significant (*P* = 0.02). Note: cp = caterpillar.

The phenotypic features and impact on virulence of a persistently activated stringent response have not been previously investigated in *S. aureus*, because of the inability to generate a mutant strain without RelA hydrolase activity [19]. Therefore, the discovery of the clinical strain JKD6229 with the active stringent response, and creation of the *relA* mutant JKD6301 provided a unique opportunity to investigate the active stringent response in this organism. A number of phenotypic characteristics were investigated (Fig. 5 and 6). JKD6301 demonstrated a reduced growth rate in MH broth, and reduced colony size on HBA after 24 hours incubation indicating that the *relA* mutation contributed significantly to the growth defect of the clinical SCV strain JKD6229 (Fig. 5A and B). An analysis of vancomycin susceptibility in JKD6301 using macromethod Etest [5], and population analysis profile (data not shown) demonstrated no increase in vancomycin resistance in the mutant compared to JKD6210 (Table 1), indicating that although the stringent response has been linked to vancomycin tolerance in *E. faecalis* [17], the *relA* mutation alone was not responsible for the reduced vancomycin susceptibility in JKD6229. Susceptibility to other antimicrobials was also unchanged in JKD6301 compared to JKD6210 (Table 1).

Previous studies have demonstrated enhanced invasion and persistence of some *S. aureus* SCV strains [6]. Therefore, the attachment, invasion, and persistence potential of the clinical isolate pair, and the *relA* mutant strain were tested (Fig. 5C, D). Bacterial attachment to HeLa cells was decreased in JKD6229 and JKD6310 compared to the parental strain JKD6210, while invasion was increased only in the SCV strain JKD6229, indicating that the stringent response promotes factors that facilitate bacterial attachment but these changes alone are not sufficient to enhance invasion. In contrast to reported studies of electron transport deficient SCV strains, after 72 hours incubation there was no difference in intracellular persistence of SCV JKD6229 compared to the other strains. This observation might reflect the activated *agr* expression and increased toxin gene expression in JKD6229 (Table 2) which is unusual for SCV *S. aureus* where reduced *agr* expression and alpha-toxin expression is thought to promote intracellular persistence without lethal effects on the host cell [7], [31], [43].

The larval stage of the Greater Wax Moth (*Galleria mellonella*) is an invertebrate model used to assess *S. aureus* virulence [44]. A comparison in this model of the virulence of parental strain JKD6210 with SCV JKD6229 and the *relA* mutant JKD6301 demonstrated a marked reduction in virulence in the SCV strain JKD6229 and also in the *relA* mutant (Fig. 6). The attenuation of SCV JKD6229 and JKD6310 was not due to their reduced growth rate compared to JKD6210 because the infected larvae had equivalent bacterial burden after 48 hours incubation. These experiments indicate that the increased persistence of SCV JKD6229 is associated with a reduced ‘virulence’ phenotype caused by the *relA* mutation.

## Discussion

In this study comparative and functional genomics has demonstrated the remarkable adaptive response of *S. aureus* to antimicrobial challenge during chronic infection, where four point mutations were sufficient to permit the strain to persist and resist multiple antibiotic therapies. This confirms the role of sequential point mutations in *S. aureus* adaptation during persistent infection, initially described by Mwangi *et al* [45]. We have uncovered a novel mechanism of growth inhibition contributing to SCV formation by *S. aureus* through mutation of *relA* and activation of the stringent response, and have described for the first time phenotypic features of an active stringent response in *S. aureus*, associated with profound global transcriptional changes. Analysis of the stringent response in *S. aureus* has been previously hampered by the inability to generate *relA* knock-out strain in this organism [21], confirming the unique nature of the naturally occurring clinical isolate JKD6229. Here, using the clinical strain JKD6229 and a mutant with a single base swap in *relA* (JKD6301), we demonstrate that an active stringent response in *S. aureus* leads to a reduced growth rate and features characteristic of SCV strains, as well as attenuated virulence in the *G. mellonella* invertebrate infection model. These data contrast with a recent report describing attenuated virulence of a *S. aureus* Rsh synthetase mutant in a murine infection model [20], suggesting that both persistent activation or inactivation of the stringent response is associated with attenuated virulence in *S. aureus*. The specific impact of the *relA* mutation was clearly demonstrated by replicating the same single nucleotide change in *relA* from the SCV strain JKD6229 into the parental strain JKD6210, and measuring the cellular levels of ppGpp using the fluorescent chemosensor PyDPA (Fig. 4). It is likely that the mutation detected in *relA* of JKD6229 partially impairs hydrolase function of the enzyme, leading to accumulation of the alarmones (p)ppGpp, but not cell death as has been described following complete loss of hydrolase function [19]. Despite the attenuated virulence of the strain in the invertebrate model, it was associated clinically with a persistent infection, suggesting that the mutation leading to permanent activation of the stringent response in this strain may have provided a survival advantage during chronic infection. Further analysis of the clinical impact of an active stringent response in *S. aureus* is now needed, with particular focus on the impact of this response on bacterial immune evasion, persistence and response to antimicrobial treatment.

In addition to an activated stringent response the clinical strain JKD6229 harboured a number of mutations leading to the reduced antimicrobial susceptibility that also promoted persistent infection. Most intriguingly, we have described for the first time a codon insertion in the methyltransferase gene SACOL1230 (RlmN) that reduces linezolid susceptibility in clinical *S. aureus*. Early reports of linezolid resistance in *S. aureus*, and other Gram positive organisms, suggested that mutations in domain V of 23S rRNA are primarily responsible for resistance [25], [26], in particular the G2576T mutation, which continues to be detected in resistant strains from multiple *S. aureus* lineages [46], [47], [48]. Recently, mutations in ribosomal proteins L3 and L4 have also been associated with linezolid resistance in staphylococci [48], [49], and it has also become apparent that changes in ribosomal methylation can affect susceptibility to linezolid and other antimicrobials in *S. aureus* and other organisms [50], [51], [52]. The conserved methyltransefrase RlmN methylates 23S rRNA at position A2503 and a *S. aureus* strain with a knock-out of the gene encoding RlmN demonstrated a 2-fold increase in linezolid susceptibility [36]. Additionally, an acquired mechanism of linezolid resistance due to acquisition of *cfr* has recently been described [53], [54]. The product of *cfr* hypermethylates 23S rRNA at position A2503 leading to the presence of not one, but two methyl groups which affects drug binding [50]. The impact of the codon insertion in SACOL1230 in our strain was confirmed by an allelic exchange experiment where the identical insertion was created in the linezolid susceptible parent strain JKD6210. Although the change in linezolid MIC was not large, this is consistent with previous reports of changes in linezolid resistance in *S. aureus* due to acquisition of the methyltransferase *cfr*, where prolonged incubation was required to detect an increase in MIC using Etest [53]. We therefore propose that the CAA insertion in SACOL1230 enhanced ribosomal methylation in the clinical isolate JKD6229 leading to a reduction in linezolid susceptibility. The clinical impact of subtle changes in linezolid susceptibility of *S. aureus* have not been defined. However, similar to recent findings with reduced vancomycin susceptibility in this organism [5], subtle reductions in susceptibility to an antibiotic may significantly impact the outcome of therapy, especially in patients with deep-seated infection as occurred in this case.

Two additional mutations were detected in JKD6229, as well as the loss of a ∼15 kb plasmid. The mutation in *rpoB* was clearly linked to the acquired rifampin resistance in JKD6229, and has been previously described [34]. Likewise, the codon insertion in *parC* contributed to an increase in quinolone MIC of the organism [35]. The plasmid which was present in JKD6210, but absent in JKD6229 (pJKD6210), shared high sequence homology to pUSA300-HOU-MS and encodes beta-lactam resistance, but did not contain the genes encoding cadmium resistance which are present on pUSA300-HOU-MS [33].

Over recent years there has been significant interest in the role of small colony variants of *S. aureus* in persisting and relapsing infections, and intracellular invasion and persistence is a frequently described feature of these strains [7]. While an understanding of the genetic determinants of SCV *S. aureus* has focussed on mutations in genes encoding hemin, menadione or thymidine biosynthesis [55], [56], [57], our data clearly demonstrates the heterogeneic nature of this phenotype, with permanent activation of the bacterial stringent response also leading to a growth defect in *S. aureus*. Not surprisingly, the global transcriptional profile and phenotypic features of stringent response *S. aureus* demonstrate significant differences to those of the defined *hemB* and *menD* mutants, while the transcriptional profile of the stringent response SCV JKD6229 shared significant similarity to the profile of *S. aureus* after exposure to mupirocin [22]. For example, auxotrophs for hemin, menadione or thymidine have been shown to have reduced tricarboxylic acid cycle (TCA) metabolism leading to reduced electron transport [8], [58], [59]. The SCV strain JKD6229 was not an auxotroph for hemin or menadione and the genes for the TCA cycle were up-regulated in JKD6229 compared to JKD6210. The SCV strain JKD6229 demonstrated increased intracellular invasion, however there was no increase in persistence compared to the parental strain (Fig. 5). Increased fibronectin-binding protein gene expression was found in JKD6229, possibly contributing to increased cellular invasion, as has been previously described for the *hemB* mutant [11]. However, the absence of increased persistence is interesting. It has previously been demonstrated that reduced *agr* expression and alpha-toxin expression occurs in clinical SCV *S. aureus*, and in the *hemB* and *medD* mutants [31], and it has been suggested that these changes favour intracellular persistence by avoiding lysis of the invaded cells [7], [43]. In the SCV strain JKD6229, increased expression of the *agr* locus was demonstrated; an unusual finding for an SCV strain, but this is also associated with the mupirocin induced stringent response in *S. aureus* [22], and could potentially explain the failure to demonstrate increased intracellular persistence. An interesting finding of this study was the profound increase in expression of capsule biosynthesis genes, associated with a significant increase in capsule production in JKD6229, demonstrated by capsule immunoblot (Fig. 2B). A previous microarray transcriptional comparison of the *hemB* mutant to its parental strain also demonstrated an increase in capsule biosynthesis genes, however not to the same degree found in JKD6229 [8]. Given the association of staphylococcal capsule production with innate immunity evasion mechanisms and virulence in animal models [60], this phenotypic change in the SCV strain JKD6229 also likely contributed to the clinical behaviour of the organism.

The growth defect of SCV JKD6229 was incompletely replicated in the *relA* mutant strain JKD6301, suggesting that additional factors contributed to the growth defect of the clinical strain. Although it appears unlikely that the other mutations detected in JKD6229 would lead to an additional growth defect, step-wise generation of each mutation in JKD6301 would be required to confirm this. Another unanswered question from this study is the mechanism of reduced vancomycin susceptibility in the strain JKD6229, which demonstrated a heterogenous-vancomycin intermediate *S. aureus* (hVISA) phenotype based on the macromethod Etest result (Table 1) [5]. Although mutations of *relA* in the Gram positive pathogen *E. faecalis* have been linked to vancomycin tolerance in that organism, there was no change in vancomycin susceptibility of the *relA* mutant JKD6301 compared to the parent strain JKD6210 demonstrating that an activated stringent response did not alter vancomycin susceptibility in this strain. Interestingly, the transcriptional profile of JKD6229 which was a hVISA, demonstrated some similarities to the transcriptional profiles of other hVISA strains, including enhanced capsule expression and reduced expression of the gene encoding protein A [30]. Finally, it is unlikely that other genomic differences were missed during our comparative genomics analysis. We performed a *de novo* assembly of the JKD6210 and JKD6229 sequences which revealed similar genome size (approx 2.7 Mb), and 97.2% coverage of the N315 genome at a depth of ≥20 for both strains. Regions of N315 not covered in the JKD6210 and JKD6229 sequences were identical, indicating that these regions were unique to N315. To reduce false positive SNP detection during comparative genomics analysis we set a threshold of a minimum depth of coverage at a SNP of ≥20, and that the reads covering that position are all uniquely and unambiguously aligned to the reference genome. Although a small possibility exists that SNPs with very low read coverage or SNPs within repeat regions might be missed in out comparative genomics analysis, this is unlikely.

In summary, using comparative and functional genomics to investigate the mechanisms of staphylococcal persistence in a patient with a very difficult-to-treat infection, we have detected a new mechanism of SCV *S. aureus*, and we have described for the first time the features of an activated stringent response in this organism. Also, a novel mechanism of reduced linezolid susceptibility has been described. Further work to determine the relationship between the stringent response and outcome of staphylococcal infections is required, as well as an exploration of the frequency of mutations in the staphylococcal gene encoding RlmN in patients treated with linezolid. This study highlights the limitations of current antimicrobial treatment strategies in patients with serious *S. aureus* infections.

## Methods

### Ethics statement

This study was performed in accordance with Austin Health Human Research Ethics Committee guidelines. The de-identified clinical details described in this manuscript constitute a medical case report that did not require formal Human Ethics Committee approval or Informed Patient Consent.

### Strains and growth conditions

Bacterial strains and plasmids used in the study are listed in Table 1. Staphylococcal strains were stored in glycerol broth at −80°C and subcultured twice onto Horse Blood Agar (Oxoid) for 48 h before being used for any experiment. Unless otherwise indicated all *S. aureus* isolates were grown in BHIB (Oxoid), and *E. coli* grown in LB broth (Oxoid). When required media was supplemented with the following antibiotics at the indicated concentrations: for *E. coli*, ampicillin 100 µg/mL; for *S. aureus* RN4220, chloramphenicol 10 µg/mL; for *S. aureus* clinical isolates, chloramphenicol 25 µg/mL. For all DNA and RNA extractions, or for experimental inoculum preparations when the SCV strain JKD6229 was used, a subculture onto solid media was performed to confirm that the strain retained the SCV phenotype. For all phenotypic experiments growth conditions were carefully controlled, and all strains were grown to the same OD_600_ prior to analysis.

### Antibiotic susceptibility and molecular typing

Vancomycin MICs were determined by microbroth MIC according to CLSI criteria [61]. The detection of vancomycin hetero-resistance was performed by macromethod Etest for vancomycin and teicoplanin as well as vancomycin population analysis, as previously described [62], [63]. A positive macromethod Etest result for hVISA was defined as vancomycin plus teicoplanin MIC≥8 µg/mL, or teicoplanin MIC≥12 µg/mL [5]. The MICs for daptomycin, gentamicin, linezolid, rifampicin and ciprofloxacin were performed by Etest (AB Biodisk), according to manufacturer's instructions. For linezolid MIC testing a 2 McFarland saline suspension was used, because of previous problems in detecting linezolid resistant strains of *S. aureus* using standard Etest [53]. Other antibiotic susceptibilities were performed by agar dilution according to CLSI criteria [61]. Pulsed-field gel electrophoresis (PFGE) and multilocus sequence typing (MLST) were also performed as previously described [62], [64].

### Growth characteristics and auxotrophism testing

Analysis of *S. aureus* growth rate was performed using 50 mL Muller Hinton II broth by inoculating 500 µL of an overnight broth culture. The optical density of the broth was read at 600 nm using a spectrometer. Assessment of colony size on solid media was performed by a blinded operator by measuring the size of 100 single colonies on Horse Blood Agar using callipers after 24 hours incubation. An analysis for hemin and menadione auxotrophism for the SCV strain JKD6229 was performed using chemically defined medium (CDM) [65] as previously described, and assessed after overnight incubation [55].

### Microarray transcriptional analysis

Microarray transcriptional analysis was performed with TIGR version 6 *S. aureus* arrays, as previously described [30]. For preparation of total RNA shaking flasks (50 mL BHI broth in 250 mL flasks) were inoculated with 500 µL overnight BHI broth culture and incubated on a 225 rpm shaker at 37°C. Optical density was closely monitored, and one millilitre of sample was collected at exponential growth phase (optical density at 600 nm of 0.5) and 0.5 mL RNA stabilization reagent (RNA later, Qiagen) was added and mixed immediately. The mixture was allowed to stand in room temperature for 10 minutes before total RNA was extracted using the RNeasy micro kit (Qiagen). RNA extractions and hybridisations were performed on four different occasions, and the dye swapped with each biological replicate. The images were combined and quantified using ImaGene™ ver 5.1 (Biodiscovery), and then imported into BASE and analyzed using Bioconductor and Limma [66], [67]. The fold ratio of gene expression for the SCV strain (JKD6229) relative to the parental MRSA (JKD6210) was calculated. Using a modified t-test P-values were calculated and adjusted for multiple testing using false discovery rate (FDR) correction. A≥2-fold change with a P value less than 0.05 was considered significant and included in an analysis of differentially expressed genes. Microarray data has been submitted to GEO with accession number GSE20957.

### Capsule polysaccharide (CP) typing and quantification

The capsule typing (CP5 and CP8) by multiplex PCR and quantification by immunoblot was performed as previously described [30]. Briefly, crude CP extracts were prepared using 10 mL of an overnight BHI broth culture adjusted to an OD_600_ of ∼0.5. Serial two-fold dilutions of CP extracts were loaded onto a nitrocellulose membrane using a dot-blot apparatus. After blocking with 5% skim milk, the membrane was hybridised with CP5-specified rabbit antiserum, hybridised with sheep anti-rabbit IgG peroxidase conjugate (Chemicon, Australia), and the image acquired and analysed using the LAS-3000 Luminescent Image Analysis System (Fujifilm, Tokyo, Japan).

### JKD6210 and JKD6229 comparative genomics

Genome sequences for the parental strain JKD6210 and the clinical SCV strain JKD6229 were obtained from an Illumina Genome Analyzer II using 36 cycle paired-end chemistry. Reads were mapped to the reference strains *S. aureus* COL (Genbank NC_002951.2) and *S. aureus* N315 (Genbank NC_002745.2) using SHRiMP. SNP/DIPs were detected using Nesoni 0.14, a software tool for analysing high-throughput DNA sequence data (http://bioinformatics.net.au/software). Nesoni tallied the raw base counts at each mapped position in each of the reference strains, and then compared them using Fisher's Exact Test to find variable nucleotide positions in JKD6229 relative to JKD6210. To exclude the possibility that mutations in JKD6229 may have occurred in regions not present in *S. aureus* COL or N315, *de novo* assembly of JKD6210 and JKD6229 was performed using Velvet 0.7.55 [68] and the above read mapping and SNP/DIP detection was performed, using the resulting contigs as reciprocal reference sequences. For SNP detection a depth of coverage of ≥20 was required at the allele. The read data for JKD6210 and JKD6229 have been deposited in the NCBI Sequence Read Archive as part of Study accession number SRP001289.

### DNA methods, molecular techniques and construction of mutants

Standard procedures were used for DNA manipulation, molecular techniques, PCR and sequencing [63], [69]. The loci containing the *relA* nucleotide substitution and the ‘CAA’ insertion in *rlmN* (from JKD6229 were amplified (Table S2), cloned with the vector pKOR1 and then generated in the parental strain JKD6210 as previously described [63]. The generation of the allele swap in JKD6210 using pJKD6318 was performed as previously described [63], with some modifications. For the final selection step, 100 µL of a 48 hour BHI broth culture (incubated at 30°C) was inoculated into 10 mL BHI broth with 5% horse blood and 400 µg/mL anhydrotetracycline. The broth was incubated for 24 hours at 37°C on a shaker at 225 rpm. The culture was then diluted to 10^−5^ and 10 µL of a range of dilutions plated on several HBA and BHI agar plates. After 24 hours incubation at 37°C, single colonies were patched on BHI agar plates with and without chloramphenicol, and screened for the correct allele swap. The correct allele swaps were confirmed, and introduction of unwanted mutations excluded, by PCR amplification and Sanger sequencing of the whole *relA* and SACOL1230 locus from the mutants strains JKD6301 and JKD6300, respectively.

### Analysis of stringent response

The presence of ppGpp was detected as previously published [42], with some modifications. Briefly, *S. aureus* strains (JKD6210, JKD6229 and JKD6301) were grown in 25 mL of BHI broth at 37°C with vigorous shaking. Serine hydroxymate (concentration 0.5 mg/mL) was added to one flask of JKD6210 for 10 minutes to induce the stringent response and provide a positive control for the assay. Cells were harvested by centrifugation at OD_600_ of 0.5. Following addition of 100% methanol, vigorous vortexing and centrifugation to pellet cellular debris, the supernatant containing ppGpp was collected and concentrated by freeze drying overnight. The dried extracts were then resuspended in 1 mM HEPES buffer, pH 7.4 containing 16% DMSO (v/v) and two-fold serial dilutions were performed in the same buffer. To each dilution, PyDPA to a final concentration of 25 µM was added. Fluorescence was observed using a hand held Wood's UV lamp (365 nm) and a FLUOstar Omega microplate reader (Ex 344 nm/Em 470 nm) (BMG Labtech, Offenburg, Germany).

### Cellular invasion and persistence assays

A HeLa cell line was used to test the invasive and intracellular persistence abilities of the clinical and mutant *S. aureus* strains. HeLa cells were seeded and grown in DMEM cell culture with 5% fetal bovin serum (FBS) in 24 well plates, and infected by the addition of approx 5×10^6^ CFU of an overnight broth culture. The correct starting inoculum was confirmed by colony counts. After 1 hour incubation at 35°C in an incubator with 5% CO_2_, the infected cells were washed with pre-warmed PBS 6 times to wash away the unattached bacteria and fresh DMEM with 5% FBS and supplemented with 400 µg/mL gentamicin and 40 µL/mL lysostaphin was added into each well and incubated for a further 72 hours. The infected HeLa cells were sampled before adding antibiotics to assess bacterial attachment/invasion, and at 1 hour post addition of antibiotics (to assess invasion), and at 24 hours and 72 hours after adding antibiotics (to assess intracellular persistence). The cell cultures were lysed by PBS supplemented with 0.05% saponin and plated on BHI agar plates. CFUs on plates were counted after 48 hr incubation at 37°C.

### *Galleria mellonella* killing assay

The previously described invertebrate *S. aureus* infection model *Galleria mellonella* [44] was used to study the pathogenesis of clinical and mutant strains. *G. mellonella* in the final instrar larval stage were used in groups of 16, and weighed to confirm no difference in size between groups. A HPLC syringe was used to inject 10 µL of bacterial suspension (approx 0.5–1.0×10^6^ CFU) into each caterpillar via the last left proleg. Bacterial colony counts were performed to confirm consistency of inoculum and caterpillars injected with PBS and caterpillars that were not injected were included as controls. Each experiment was repeated on at least 4 different occasions. To determine the bacterial burden in infected caterpillars 48 hours after inoculation an assessment of the *S. aureus* CFU per caterpillar was performed on a subset of caterpillars.

### Statistical analysis

Non-parametric tests were used to analyse the results of colony size, bacterial attachment, invasion and persistence assays. Statistical analyses were performed using the two-tailed Mann-Whitney *U* test, with a *p*<0.05 set for statistical significance. Growth curves and stringent response activity were analysed using a one way analysis of variance (ANOVA) at each time point, and Kaplan Meier plots of *G. mellonella* killing results were analysed using the log rank test. All analyses were performed using Prism 4 for Macintosh ver 4.0 (GraphPad Software Inc., CA, USA).

## Supporting Information

Table S1 Microarray transcriptional results for JKD6229 compared to JKD6210 with complete list of differentially regulated genes.
(0.23 MB PDF)

Table S2 Primers used in this study.
(0.02 MB PDF)
