# Supplementary material for: Two Novel Point Mutations in Clinical Staphylococcus aureus Reduce Linezolid Susceptibility and Switch on the Stringent Response to Promote Persistent Infection
Source: PLoS Pathog. 2010 Jun 10;6(6):e1000944. doi: 10.1371/journal.ppat.1000944 (PMC2883592; doi:10.1371/journal.ppat.1000944)
Supplement: Table S1 — Microarray transcriptional results for JKD6229 compared to JKD6210 with complete list of differentially regulated genes. (0.23 MB PDF) [file ppat.1000944.s001.pdf]

Table S1. Microarray Transcriptional Results for JKD6229 compared to JKD6210.				
Gene ID	Gene Name	Gene Product	adjusted P value	Fold ratio
DOWN-Regulated				
MW2338		hypothetical protein	0.00	0.18
pSAS12	*cadC*	putative cadmium efflux system regulatory protein	0.03	0.19
pSAS17	*blaI*	penicillinase repressor	0.00	0.10
SA0210		hypothetical protein	0.00	0.44
SACOL0045		hypothetical protein	0.00	0.22
SACOL0190	*acpD*	acyl carrier protein phosphodiesterase	0.00	0.28
SACOL0199		hypothetical protein	0.00	0.46
SACOL0433		hypothetical protein	0.00	0.34
SACOL0610	*sdrE*	sdrE protein	0.01	0.43
SACOL1066		fmt protein	0.00	0.49
SACOL1454		acetyltransferase, GNAT family	0.00	0.27
SACOL1847		conserved domain protein, putative	0.00	0.31
SACOL1907		ribosomal large subunit pseudouridine synthase, RluD subfamily	0.00	0.41
SACOL2180	*lacG*	6-phospho-beta-galactosidase	0.00	0.03
SACOL2381		hypothetical protein	0.00	0.23
SACOL2400		acetyltransferase, GNAT family	0.00	0.37
SACOL2487		hypothetical protein	0.00	0.49
SACOL2695		hypothetical protein	0.01	0.45
SAR0219		hypothetical protein	0.00	0.16
SAR0318		hypothetical protein	0.05	0.49
SAR0400		nitroreductase family protein	0.00	0.44
SAR0419		hypothetical protein	0.03	0.48
SAR0477	*dnaX*	DNA polymerase III, tau subunit	0.04	0.42
SAR0686		pseudo	0.03	0.30
SAR0706		hypothetical protein	0.00	0.22
SAR0719		putative resolvase	0.03	0.37
SAR1949		putative extracellular glutamine-binding protein	0.05	0.40
SAR2198	*atpI*	putative ATP synthase protein I	0.01	0.47
SAR2281	*lacE*	PTS system, lactose-specific IIBC component	0.01	0.35
SAR2502		ABC transporter ATP-binding protein	0.00	0.36
SAR2697		precorrin-2 dehydrogenase	0.02	0.45
SAR2755	*hisF*	HisF cyclase-like protein	0.01	0.30
SAR2757	*hisH*	putative amidotransferase	0.02	0.21
SAS0082		putative myosin-crossreactive antigen	0.00	0.23
SAS0164		glucose-specific PTS transporter protein, IIABC component	0.00	0.22
SAS0203		hypothetical protein	0.00	0.16
SAS0214		hypothetical protein	0.00	0.20
SAS0215		hypothetical protein	0.00	0.19
SAS0216		flavohemoprotein	0.00	0.13
SAS0236		cell wall metabolism protein	0.00	0.09
SAS0383		hypothetical protein	0.00	0.27
SAS0387		exotoxin	0.00	0.49
SAS1719		putative lipoprotein	0.00	0.21
SAS1838		putative oxygenase	0.00	0.28
SAS2090		6-phospho-beta-galactosidase	0.00	0.04
SAS2096		galactose-6-phosphate isomerase	0.00	0.01
SAS2341		hypothetical protein	0.00	0.41
SAS2358		oligopeptide transporter putative substrate binding domain	0.00	0.29
SAS2410		2-hydroxyacid dehydrogenase	0.00	0.48
SAS2532		putative surface anchored protein	0.00	0.48
SAV0032		hypothetical protein	0.00	0.33
SAV0039		glycerophosphoryl diester phosphodiesterase homolog	0.01	0.48
SAV0059		hypothetical protein	0.05	0.42
SAV0105		hypothetical protein	0.01	0.42
SAV0106		67 kDa Myosin-crossreactive streptococcal antigen homolog	0.00	0.22
SAV0111	*spa*	Immunoglobulin G binding protein A precursor	0.00	0.42
SAV0148	*adhE*	alcohol-acetaldehyde dehydrogenase	0.04	0.29
SAV0189	*glcA*	PTS enzyme II	0.00	0.18
SAV0218		similar to NADH-dependent dehydrogenase	0.00	0.45
SAV0226	*pflB*	formate acetyltransferase	0.00	0.16
SAV0227	*pflA*	formate acetyltransferase activating enzyme	0.00	0.13
SAV0237		similar to nickel ABC transporter nickel-binding protein	0.02	0.30
SAV0240		putative flavohemoprotein	0.00	0.13
SAV0242		PTS enzyme	0.00	0.11
SAV0254		trancated TagF	0.01	0.49
SAV0259	*scdA*	cell division and morphogenesis-related protein	0.00	0.15
SAV0262	*lrgA*	murein hydrolase regulator LrgA	0.00	0.13
SAV0321		hypothetical protein	0.00	0.34
SAV0337	*glpT*	glycerol-3-phosphate transporter	0.00	0.43
SAV0354		acetyl-CoA C-acetyltransferase homolog	0.00	0.46
SAV0362		hypothetical protein	0.00	0.47
SAV0363		GTP-binding protein	0.00	0.39
SAV0382		similar to nitro/flavin reductase	0.00	0.42
SAV0397		putative transcriptional regulator	0.03	0.38
SAV0420		hypothetical protein	0.00	0.31
SAV0421		similar to oxidoreductase	0.00	0.28
SAV0433	*set15*	exotoxin 15	0.01	0.32
SAV0434		hypothetical protein	0.00	0.21
SAV0521	*nupC*	pyrimidine nucleoside transport protein	0.00	0.33
SAV0525	*clpC*	endopeptidase	0.00	0.48
SAV0562	*sdrD*	Ser-Asp rich fibrinogen-binding, bone sialoprotein-binding protein	0.00	0.28
SAV0563	*sdrE*	Ser-Asp rich fibrinogen-binding, bone sialoprotein-binding protein	0.00	0.40
SAV0587		hypothetical protein	0.02	0.48
SAV0605	*adh1*	alcohol dehydrogenase	0.02	0.44
SAV0807		hypothetical protein	0.02	0.43
SAV0808		hypothetical protein	0.02	0.46
SAV0945		hypothetical protein	0.00	0.08
SAV0962	*pgi*	glucose-6-phosphate isomerase	0.00	0.33
SAV0975	*clpB*	ClpB chaperone homolog	0.01	0.39
SAV0983	*fabH*	3-oxoacyl-(acyl carrier protein) synthase	0.00	0.45

SAV1037		hypothetical protein	0.00	0.34
SAV1086		cytochrome D ubiquinol oxidase subunit 1 homolog	0.00	0.25
SAV1087		cytochrome D ubiquinol oxidase subunit II homolog	0.00	0.27
SAV1123		hypothetical protein	0.00	0.39
SAV1205	*pyrE*	orotate phosphoribosyltransferase	0.02	0.13
SAV1206		hypothetical protein	0.04	0.30
SAV1287		hypothetical protein	0.01	0.38
SAV1306	*bsaA*	glutathione peroxidase	0.00	0.34
SAV1316		hypothetical protein	0.03	0.43
SAV1336	*rpsN*	30S ribosomal protein S14	0.00	0.32
SAV1338		hypothetical protein	0.01	0.33
SAV1347	*mscL*	large-conductance mechanosensitive channel	0.00	0.43
SAV1360	*fmtC*	oxacillin resistance-related FmtC protein	0.00	0.31
SAV1362	*msrR*	peptide methionine sulfoxide reductase regulator	0.00	0.26
SAV1363		4-oxalocrotonate tautomerase	0.00	0.38
SAV1374	*femA*	factor essential for expression of methicillin resistance	0.00	0.50
SAV1417		hypothetical protein	0.01	0.47
SAV1418	*murG*	N-acetylglucosaminyl transferase	0.00	0.22
SAV1419		hypothetical protein	0.00	0.32
SAV1436		Blt-like protein	0.00	0.02
SAV1437		similar to amino acid permease family protein	0.00	0.03
SAV1438		threonine dehydratase	0.00	0.03
SAV1439		alanine dehydrogenase	0.00	0.01
SAV1442		similar to sulfite reductase [NADPH] flavoprotein	0.00	0.08
SAV1478	*cmk*	cytidylate kinase	0.00	0.46
SAV1730	*sgtA*	probable transglycosylase	0.03	0.34
SAV1799		hypothetical protein	0.00	0.22
SAV1800		hypothetical protein	0.00	0.23
SAV1834	*hemE*	uroporphyrinogen decarboxylase	0.00	0.50
SAV1850		hypothetical protein	0.00	0.41
SAV1857		glutamate ABC transporter ATP-binding protein	0.02	0.25
SAV1948	*sep*	enterotoxin P	0.00	0.38
SAV2030	*groES*	GroES protein	0.00	0.41
SAV2090		lipoprotein precursor	0.00	0.44
SAV2130		hypothetical protein	0.00	0.37
SAV2136	*pdp*	pyrimidine-nucleoside phosphorylase	0.00	0.34
SAV2189	*lacG*	6-phospho-beta-galactosidase	0.00	0.01
SAV2190	*lacE*	PTS system, lactose-specific IIBC component	0.00	0.02
SAV2191	*lacF*	PTS system, lactose-specific IIA component	0.00	0.02
SAV2192	*lacD*	tagatose 1,6-diphosphate aldolase	0.00	0.01
SAV2193	*lacC*	tagatose-6-phosphate kinase	0.00	0.01
SAV2194	*lacB*	galactose-6-phosphate isomerase	0.00	0.03
SAV2256		glucose uptake protein homolog	0.00	0.46
SAV2279	*modA*	probable molybdate-binding protein	0.00	0.36
SAV2324		transcriptional regulator	0.02	0.44
SAV2353		similar to multidrug resistance protein A	0.00	0.50
SAV2370		probable oxidoreductase	0.00	0.26
SAV2373		hypothetical protein	0.00	0.17
SAV2402		hypothetical protein	0.00	0.37
SAV2403		similar to NirC protein	0.00	0.23
SAV2406		similar to Zn-binding lipoprotein adcA	0.00	0.37
SAV2458		hypothetical protein	0.02	0.38
SAV2463		oligopeptide transporter putative ATPase domain	0.00	0.47
SAV2464		oligopeptide transporter putative ATPase domain	0.05	0.48
SAV2465		oligopeptide transporter putative membrane permease domain	0.00	0.42
SAV2466		oligopeptide transporter putative membrane permease domain	0.00	0.32
SAV2467		oligopeptide transporter putative substrate binding domain	0.00	0.31
SAV2477		hypothetical protein	0.00	0.48
SAV2478		similar to oxidoreductase	0.00	0.31
SAV2523		similar to NAD(P)H-flavin oxidoreductase	0.00	0.45
SAV2538	*ptsG*	PTS system, glucose-specific II ABC component	0.01	0.30
SAV2547	*adaB*	probable methylated DNA-protein cysteine methyltransferase	0.00	0.46
SAV2555		putative acetyltransferase	0.04	0.39
SAV2557	*copA*	copper-transporting ATPase	0.00	0.46
SAV2558		similar to mercuric ion-binding protein	0.00	0.44
SAV2582		similar to cobalamin synthesis related protein CobW	0.01	0.43
SAV2583		similar to ferrous iron transporter protein B	0.01	0.24
SAV2584		probable monooxygenase	0.00	0.47
SAV2589		dihydroorotate dehydrogenase	0.00	0.12
SAV2591		hypothetical protein	0.00	0.50
SAV2600		2-dehydropantoate 2-reductase	0.00	0.34
SAV2602		L-lactate dehydrogenase	0.00	0.30
SAV2616		anaerobic (class III) ribonucleotide reductase small subunit chain	0.00	0.32
SAV2617	*nrdD*	anaerobic ribonucleoside triphosphate reductase	0.01	0.30
SAV2632	*arcC*	carbamate kinase	0.00	0.43
SAV2638	*isaB*	immunodominant antigen B	0.00	0.38
SAV2646		hypothetical protein	0.00	0.49
SAV2675	*hisH*	amidotransferase hisH	0.02	0.31
SAV2676	*hisB*	imidazoleglycerol-phosphate dehydratase	0.04	0.35
SAV2678		Histidinol dehydrogenase	0.02	0.31
SAV2680		probable ATP phosphoribosyltransferase regulatory subunit	0.02	0.12
SAV2687		hypothetical protein	0.00	0.45
SAV2707		hypothetical protein	0.00	0.43
SAVP016		hypothetical protein	0.00	0.21
SAVP034		replication-associated protein	0.00	0.15
**UP-Regulated**				
MW0040		hypothetical protein	0.03	2.59
MW0418		hypothetical protein	0.03	2.37
MW1357		hypothetical protein	0.00	5.10
MW1411		hypothetical protein	0.00	12.85
MW1413		hypothetical protein	0.00	32.98
MW1415		hypothetical protein	0.01	3.58

MW1419		hypothetical protein	0.01	3.49
MW1430		hypothetical protein	0.00	41.07
MW1895		hypothetical protein	0.00	12.66
MW1917		hypothetical protein	0.00	25.25
MW1932		phage anti repressor	0.00	23.29
MW2435		fructose-bisphosphatase (fbp)	0.00	2.15
SA0145	*capB*	capsular polysaccharide synthesis enzyme Cap5B	0.00	54.01
SA1245	*odhA*	2-oxoglutarate dehydrogenase E1	0.01	2.03
SA1753		hypothetical protein	0.02	2.08
SA1754		hypothetical protein	0.00	3.57
SA1759		lytic enzyme	0.00	6.31
SA1761	*sep*	enterotoxin P	0.00	2.76
SA1765		hypothetical protein	0.00	34.33
SA1766		hypothetical protein	0.00	29.91
SA1770		hypothetical protein	0.00	56.47
SA1779		hypothetical protein	0.00	37.14
SA1781		hypothetical protein	0.00	20.94
SA1782		hypothetical protein	0.00	34.00
SA1783		hypothetical protein	0.00	37.53
SA1784		hypothetical protein	0.00	21.46
SA1785		hypothetical protein	0.00	19.95
SA1786		hypothetical protein	0.00	24.90
SA1787		hypothetical protein	0.00	21.31
SA1788		hypothetical protein	0.00	20.70
SA1789		hypothetical protein	0.00	56.15
SA1790		hypothetical protein	0.00	15.51
SA1792		single-strand DNA-binding protein	0.00	53.14
SA1793		hypothetical protein	0.00	52.48
SA1794		hypothetical protein	0.00	58.47
SA1795		hypothetical protein	0.00	73.61
SA1796		hypothetical protein	0.00	8.40
SA1797		hypothetical protein	0.00	21.57
SA1798		hypothetical protein	0.00	21.91
SA1799		hypothetical protein	0.00	43.26
SA1802		hypothetical protein	0.01	5.46
SA1803		hypothetical protein	0.00	41.28
SA1804		hypothetical transcriptional regulator	0.00	19.79
SA1805		hypothetical protein	0.00	4.78
SA1808		probable ss-1,3-N-acetylglucosaminyltransferase	0.00	2.45
SA1809		hypothetical protein	0.00	3.39
SA1810	*int*	integrase	0.00	3.45
SA2445		hypothetical protein	0.00	4.19
SACOL0012		homoserine O-acetyltransferase, putative	0.03	2.78
SACOL0035		hypothetical protein	0.00	4.85
SACOL0334		hypothetical protein	0.00	46.37
SACOL0351		hypothetical protein	0.00	65.42
SACOL0356		hypothetical protein	0.00	43.20
SACOL0390		lipase precursor, interruption-C	0.00	2.48
SACOL0790.1		ribonucleoside-diphosphate reductase 2, NrdH-redoxin, putative	0.03	2.39
SACOL0919		hypothetical protein	0.00	3.54
SACOL1165		hypothetical protein	0.01	2.05
SACOL1186		antibacterial protein (phenol soluble modulin)	0.00	7.55
SACOL1187		antibacterial protein (phenol soluble modulin)	0.00	2.03
SACOL1832		crcB protein (related to Genetic Information Processing)	0.00	2.37
SACOL1877	*epiB*	epidermin biosynthesis protein EpiB	0.00	6.53
SACOL1999		hypothetical protein	0.00	9.98
SACOL2003	*hlb*	phospholipase C	0.01	2.06
SACOL2065		hypothetical protein	0.00	2.76
SACOL2069	*kdpF*	K+-transporting ATPase, F subunit	0.02	3.64
SACOL2505		cell wall surface anchor family protein	0.00	10.06
SACOL2676		LPXTG cell wall surface anchor family protein	0.02	2.33
SACOL2678		hypothetical protein	0.02	2.42
SAR0151	*capA*	capsular polysaccharide synthesis enzyme	0.00	41.00
SAR0226	*fadE*	putative acyl-CoA synthetase	0.03	2.76
SAR0339		putative acetyltransferase	0.00	2.38
SAR0378		hypothetical protein	0.00	4.91
SAR0380		hypothetical protein	0.00	3.39
SAR0381		hypothetical protein	0.00	3.80
SAR0445		putative lipoprotein	0.01	2.00
SAR0665		putative esterase	0.02	3.05
SAR0953		transport system extracellular binding lipoprotein	0.03	2.57
SAR1563		pseudo	0.00	22.46
SAR2031		pseudo	0.01	3.64
SAR2051		hypothetical protein	0.00	21.09
SAR2052		hypothetical protein	0.00	16.88
SAR2053		hypothetical protein	0.00	8.16
SAR2065a		hypothetical protein	0.00	43.37
SAR2076		hypothetical protein	0.00	25.23
SAR2077		hypothetical protein	0.00	9.76
SAR2087		hypothetical protein	0.00	66.27
SAR2090		hypothetical protein	0.01	4.58
SAR2091		hypothetical protein	0.00	13.43
SAR2096		putative anti repressor	0.00	52.51
SAR2150		hypothetical protein	0.00	2.48
SAR2151		putative RNA binding protein	0.00	2.28
SAR2239		hypothetical protein	0.00	7.71
SAR2384		hypothetical protein	0.00	4.35
SAS0028		hypothetical protein	0.00	2.39
SAS0360		putative sodium:dicarboxylate symporter protein	0.00	2.45
SAS0368		hypothetical protein	0.01	2.15
SAS0419		ABC transporter ATP-binding protein	0.00	3.08
SAS0431		sugar-specific PTS transport system, IIBC component	0.00	2.24
SAS063		hypothetical protein	0.00	60.91
SAS0630		hypothetical protein	0.01	2.73

SAS064		hypothetical protein	0.00	22.61
SAS0903		hypothetical protein	0.02	3.64
SAS0912		hypothetical protein	0.00	15.37
SAS0913		hypothetical protein	0.00	60.16
SAS1076		hypothetical protein	0.02	2.03
SAS1090		hypothetical protein	0.00	3.33
SAS1103		carbamate kinase	0.02	2.44
SAS1448		maltose operon transcriptional repressor	0.00	7.54
SAS1545		hypothetical protein	0.00	4.46
SAS1622		isocitrate dehydrogenase	0.00	3.21
SAS1760a		hypothetical protein	0.00	3.33
SAS1804		hypothetical protein	0.00	2.02
SAS1903		putative phage regulatory protein	0.00	23.73
SAS1907		hypothetical protein	0.00	3.95
SAS1908		hypothetical protein	0.00	96.54
SAS1910		hypothetical protein	0.00	56.94
SAS1936		membrane anchored protein	0.00	3.59
SAS1940a		delta-hemolysin precursor	0.00	10.29
SAS2077		FecCD transport family protein	0.00	3.33
SAS2104		hypothetical protein	0.02	2.31
SAS2362		hypothetical protein	0.00	5.07
SAS2376		putative helicase	0.00	69.80
SAS2401		hypothetical protein	0.00	2.15
SAS2401a		hypothetical protein	0.04	2.71
SAS2490		hypothetical protein	0.03	2.74
SAV0041	*mecA*	penicillin binding protein 2 prime	0.00	25.98
SAV0042	*mecR1*	methicillin resistance protein	0.00	7.69
SAV0110	*lctP*	L-lactate permease homolog	0.00	2.59
SAV0146		hypothetical protein	0.00	2.14
SAV0147		similar to transcription regulator	0.00	2.06
SAV0149	*capA*	capsular polysaccharide synthesis enzyme Cap5A	0.00	13.27
SAV0150	*capB*	capsular polysaccharide synthesis enzyme Cap5B	0.00	10.93
SAV0151	*capC*	capsular polysaccharide synthesis enzyme Cap8C	0.00	12.01
SAV0152	*capD*	capsular polysaccharide synthesis enzyme Cap5D	0.00	37.31
SAV0152	*capD*	capsular polysaccharide synthesis enzyme Cap5D	0.00	60.21
SAV0153	*capE*	capsular polysaccharide synthesis enzyme Cap8E	0.00	25.69
SAV0154	*capF*	capsular polysaccharide synthesis enzyme Cap5F	0.00	80.66
SAV0155	*capG*	capsular polysaccharide synthesis enzyme Cap5G	0.00	22.98
SAV0156	*capH*	capsular polysaccharide synthesis enzyme O-acetyl transferase Cap5I	0.00	19.04
SAV0157	*capI*	capsular polysaccharide synthesis enzyme Cap5I	0.00	39.32
SAV0158	*capJ*	capsular polysaccharide synthesis enzyme Cap5J	0.00	14.52
SAV0159	*capK*	capsular polysaccharide synthesis enzyme Cap5K	0.00	10.34
SAV0160	*capL*	capsular polysaccharide synthesis enzyme Cap5L	0.00	7.16
SAV0161	*capM*	capsular polysaccharide synthesis enzyme Cap5M	0.00	4.56
SAV0162	*capN*	capsular polysaccharide synthesis enzyme Cap5N	0.00	11.92
SAV0163	*capO*	capsular polysaccharide synthesis enzyme Cap8O	0.00	2.94
SAV0167	*aldA*	aldehyde dehydrogenase homolog	0.00	2.40
SAV0173		hypothetical protein	0.00	2.26
SAV0176		hypothetical protein	0.00	2.39
SAV0178		similar to integral membrane protein LmrP	0.00	3.22
SAV0179		similar to surfactin synthetase	0.01	4.86
SAV0180		hypothetical protein	0.02	2.26
SAV0183	*argJ*	bifunctional ornithine acetyltransferase/N-acetylglutamate synthase	0.01	2.61
SAV0185		ornithine aminotransferase	0.01	2.69
SAV0205	*oppF*	oligopeptide transport ATP-binding protein	0.00	4.54
SAV0234		putative acyl-CoA synthetase FadE	0.01	5.50
SAV0235		putative acetyl-CoA/acetoacetyl-CoA transferase	0.00	12.52
SAV0247	*gatC*	probable PTS galactitol-specific enzyme IIC component	0.03	2.00
SAV0320	*geh*	glycerol ester hydrolase	0.00	3.55
SAV0340		similar to NADH-dependent FMN reductase	0.02	2.63
SAV0342		similar to ribosomal-protein-serine N-acetyltransferase	0.00	2.81
SAV0364		hypothetical protein	0.01	2.58
SAV0377		hypothetical protein	0.00	2.56
SAV0388	*xprT*	xanthine phosphoribosyltransferase	0.02	2.63
SAV0389	*pbuX*	xanthine permease	0.00	2.99
SAV0410		hypothetical protein	0.05	2.74
SAV0416		hypothetical protein	0.01	2.50
SAV0462		similar to ABC transporter ATP-binding protein	0.00	3.07
SAV0463		ABC transporter permease protein	0.00	6.67
SAV0553		UDP-glucose 4-epimerase related protein	0.00	2.44
SAV0584		cationic amino acid transporter	0.00	2.33
SAV0621		putative NADH dehydrogenase I chain L	0.03	2.27
SAV0626		Na_ antiporter	0.00	2.47
SAV0628		hypothetical protein	0.00	2.37
SAV0661	*vraF*	ABC transporter ATP-binding protein	0.00	2.54
SAV0667		similar to AraC/XylS family transcriptional regulator	0.05	2.10
SAV0692		hypothetical protein	0.00	3.09
SAV0693		hypothetical protein	0.00	3.13
SAV0695	*norA*	quinolone resistance protein	0.00	2.98
SAV0696		hypothetical protein	0.01	2.03
SAV0698		similar to transcription repressor of fructose operon	0.00	2.59
SAV0700	*fruA*	fructose specific permease	0.00	2.17
SAV0732	*nrdF*	ribonucleotide-diphosphate reductase beta subunit	0.00	2.00
SAV0758	*uvrB*	excinuclease ABC subunit B	0.00	5.42
SAV0759	*uvrA*	exinuclease ABC subunit A	0.00	2.63
SAV0809		hypothetical protein	0.00	2.02
SAV0811	*fnb*	fibrinogen-binding protein	0.00	2.13
SAV0819		hypothetical protein	0.00	2.16
SAV0820		hypothetical protein	0.00	5.24
SAV0821		hypothetical protein	0.01	4.09
SAV0837		ABC transporter ATP-binding protein homolog	0.00	3.01
SAV0838		similar to ABC transporter, permease protein homolog	0.00	4.03
SAV0839		similar to ABC transporter substrate-binding protein	0.00	6.88
SAV0875		phi PVL ORF 52 homolog	0.00	54.57

SAV0876		similar to phi ETA orf 34-like protein	0.00	38.57
SAV0877		hypothetical protein	0.00	13.21
SAV0957	*rocD*	ornithine--oxo-acid transaminase	0.00	2.64
SAV0986	*oppB*	oligopeptide transport system permease protein	0.00	2.53
SAV0987		oligopeptide transport system permease protein OppC	0.00	4.94
SAV0988	*oppD*	oligopeptide transport system ATP-binding protein OppD homolog	0.00	7.27
SAV0989	*oppF*	oligopeptide transport system ATP-binding protein OppF homolog	0.00	7.36
SAV0990		similar to peptide binding protein OppA	0.00	6.06
SAV0994	*oppB*	probable oligopeptide transport system permease protein	0.00	3.19
SAV1023	*htrA*	serine protease HtrA	0.00	3.34
SAV1036		hypothetical protein	0.00	2.90
SAV1049		HisC homolog	0.00	3.07
SAV1052		partial autolysin	0.00	2.55
SAV1054		acetyl transferase	0.00	2.08
SAV1147	*sdhC*	succinate dehydrogenase cytochrome b-558	0.00	9.35
SAV1148	*sdhA*	succinate dehydrogenase	0.00	2.92
SAV1163		alpha-hemolysin precursor	0.00	5.94
SAV1187		hypothetical protein	0.00	2.16
SAV1191		YlmH	0.00	2.72
SAV1300	*glpF*	glycerol uptake facilitator	0.00	2.08
SAV1408		hypothetical protein	0.03	3.74
SAV1413	*odhA*	oxoglutarate dehydrogenase	0.00	2.12
SAV1497	*xerD*	site-specific recombinase	0.00	2.14
SAV1507	*malA*	alpha-D-1,4-glucosidase	0.00	5.73
SAV1508	*malR*	maltose operon transcriptional repressor	0.00	7.98
SAV1535		glycine dehydrogenase subunit 2	0.00	2.36
SAV1536		glycine dehydrogenase subunit 1	0.00	2.53
SAV1537		aminomethyltransferase	0.00	3.29
SAV1559		hypothetical protein	0.00	2.48
SAV1588		similar to late competence protein ComEC	0.00	2.35
SAV1589	*comEB*	late competence operon required for DNA binding and uptake	0.00	2.26
SAV1604		probable transmembrane transport protein	0.02	2.79
SAV1605		hypothetical protein	0.00	3.01
SAV1606		acetyl-CoA carboxylase	0.00	2.71
SAV1607		similar to acetyl-CoA carboxylase (biotin carboxyl carrier subunit)	0.00	2.87
SAV1608		hypothetical protein	0.00	2.71
SAV1610	*greA*	transcription elongation factor	0.00	2.92
SAV1611	*udk*	uridine kinase	0.01	2.17
SAV1612		protease	0.00	2.08
SAV1613		protease	0.00	3.18
SAV1614		similar to caffeoyl-CoA O-methyltransferase	0.00	3.77
SAV1619		similar to deoxyribonuclease	0.00	2.29
SAV1643		hypothetical protein	0.01	2.03
SAV1665		hypothetical protein	0.00	2.00
SAV1687	*gapB*	glyceraldehyde 3-phosphate dehydrogenase 2	0.02	2.05
SAV1695	*citZ*	citrate synthase	0.00	5.79
SAV1723		similar to transaminase	0.01	3.09
SAV1732	*fhs*	formyltetrahydrofolate synthetase	0.01	2.27
SAV1733	*acsA*	acetyl-coenzyme A synthetase	0.00	4.12
SAV1783		hypothetical protein	0.00	2.37
SAV1791	*pckA*	phosphoenolpyruvate carboxykinase	0.00	2.29
SAV1812	*splB*	serine protease	0.01	2.26
SAV1816		truncated hypothetical protein	0.00	2.34
SAV1852		hypothetical protein	0.00	3.21
SAV1860	*tnp*	transposase	0.01	2.26
SAV1883		similar to transporter	0.00	2.43
SAV1896		hypothetical protein	0.02	2.45
SAV1916		similar to sodium-dependent transporter	0.00	3.98
SAV1922		hypothetical protein	0.00	6.29
SAV1941		hypothetical protein	0.02	4.27
SAV1946		holin homolog	0.00	4.74
SAV1947		hypothetical protein	0.00	2.00
SAV1950		hypothetical protein	0.00	11.81
SAV1951		similar to phi PVL ORF 22 homolog	0.00	8.62
SAV1952		hypothetical protein	0.00	8.88
SAV1957		hypothetical protein	0.00	45.94
SAV1958		hypothetical protein	0.00	24.06
SAV1960		hypothetical protein	0.00	12.52
SAV1961		hypothetical protein	0.00	9.91
SAV1962		hypothetical protein	0.00	25.59
SAV1963		hypothetical protein	0.00	26.59
SAV1964		phiN315 scaffolding protein-like protein	0.00	22.00
SAV1965		hypothetical protein	0.00	33.98
SAV1966		phage terminase large subunit	0.00	14.52
SAV1967		hypothetical protein	0.00	17.33
SAV1969		hypothetical protein	0.00	12.40
SAV1971		hypothetical protein	0.00	15.88
SAV1972		hypothetical protein	0.00	19.09
SAV1978		PVL orf 51-like protein	0.00	13.50
SAV1979		phi PVL ORF 50 homolog	0.00	7.67
SAV1982		hypothetical protein	0.00	72.92
SAV1983		single-strand DNA-binding protein	0.00	20.38
SAV1990		phi PVL ORF 38 homolog	0.00	34.49
SAV1991		hypothetical protein	0.00	12.38
SAV2000		hypothetical protein	0.02	2.02
SAV2004		hypothetical protein	0.05	2.25
SAV2012		similar to bacteriophage terminase small subunit	0.00	3.29
SAV2032		hypothetical protein	0.00	3.89
SAV2036	*agrB*	accessory gene regulator B	0.00	2.99
SAV2037	*agrD*	AgrD protein	0.00	2.22
SAV2038	*agrC*	accessory gene regulator C	0.00	6.43
SAV2039	*agrA*	accessory gene regulator A	0.00	2.80
SAV2045		probable transport system permease protein	0.00	2.25
SAV2053	*ilvD*	dihydroxy-acid dehydratase	0.02	6.03

SAV2054	*ilvB*	acetolactate synthase large subunit	0.04	4.54
SAV2057	*leuA*	2-isopropylmalate synthase	0.01	7.07
SAV2058	*leuB*	3-isopropylmalate dehydrogenase	0.03	2.58
SAV2059	*leuC*	isopropylmalate isomerase large subunit	0.01	4.81
SAV2060	*leuD*	3-isopropylmalate dehydratase small subunit	0.00	3.32
SAV2062		hypothetical protein	0.01	2.01
SAV2076	*kdpB*	probable potassium-transporting ATPase B chain	0.00	3.30
SAV2078	*kdpD*	sensor protein	0.00	2.11
SAV2115		similar to phosphatase	0.03	2.49
SAV2116		hypothetical protein	0.00	2.19
SAV2133	*hmrA*	HmrA	0.02	2.00
SAV2145	*czrA*	repressor protein	0.00	2.03
SAV2146	*czrB*	cation-efflux system membrane protein homolog	0.00	3.11
SAV2148		hypothetical protein	0.00	2.55
SAV2150		hypothetical protein	0.01	3.81
SAV2151		hypothetical protein	0.00	2.33
SAV2166		similar to multidrug resistance protein	0.00	2.52
SAV2169		probable multidrug transporter	0.01	2.47
SAV2175	*htsC*	heme transport system permease	0.00	4.81
SAV2176	*htsB*	heme transport system permease	0.00	5.02
SAV2180		transporter	0.00	2.35
SAV2297		hypothetical protein	0.00	2.81
SAV2298		similar to transcription regulator	0.00	7.83
SAV2299	*ssaA*	secretory antigen precursor SsaA homolog	0.01	2.43
SAV2300		hypothetical protein	0.00	3.10
SAV2301		similar to Na_ antiporter	0.00	4.53
SAV2301		similar to Na_ antiporter	0.00	5.17
SAV2302		D-octopine dehydrogenase	0.00	3.16
SAV2304		hypothetical protein	0.00	4.13
SAV2327		hypothetical protein	0.00	2.56
SAV2332		hypothetical protein	0.01	2.00
SAV2377	*scrA*	PTS system, sucrose-specific IIBC component	0.00	2.95
SAV2380		hypothetical protein	0.02	4.51
SAV2381		similar to transcription regulatory protein	0.02	3.56
SAV2422		hypothetical protein	0.03	2.07
SAV2437		probable phosphoesterase	0.01	2.12
SAV2440		similar to amino acid permease	0.02	2.11
SAV2441		fmtA-like protein	0.00	2.97
SAV2473		similar to aminobenzoyl-glutamate transport protein	0.00	2.95
SAV2481		hypothetical protein	0.00	2.24
SAV2495		hypothetical protein	0.01	5.42
SAV2496		hypothetical protein	0.00	10.06
SAV2497		similar to accumulation-associated protein (truncated)	0.00	20.31
SAV2502	*fnbB*	fibronectin-binding protein homolog	0.01	2.37
SAV2503	*fnb*	fibronectin-binding protein homolog	0.02	2.81
SAV2506	*gntK*	gluconokinase	0.00	8.45
SAV2507	*gntR*	gluconate operon transcriptional repressor	0.00	4.07
SAV2516	*fbp*	fructose-bisphosphatase	0.00	3.55
SAV2540		hypothetical protein	0.00	6.64
SAV2541		hypothetical protein	0.00	3.65
SAV2544		similar to secretory antigen precursor SsaA	0.00	6.41
SAV2559		2-hydroxyacid dehydrogenase	0.00	2.46
SAV2560		N-succinyldiaminopimelate aminotransferase	0.00	2.54
SAV2564		similar to phytoene dehydrogenase	0.00	2.05
SAV2565		hypothetical protein	0.00	2.84
SAV2566		similar to secretory antigen precursor SsaA	0.00	2.37
SAV2571		hypothetical protein	0.00	3.46
SAV2651		hypothetical protein	0.00	2.14
SAV2653		similar to preprotein translocase secY	0.02	2.90
SAV2667	*icaD*	intercellular adhesion protein D	0.00	2.63
SAVP007		hypothetical protein	0.00	2.64
