# Supplementary material for: Two Novel Point Mutations in Clinical Staphylococcus aureus Reduce Linezolid Susceptibility and Switch on the Stringent Response to Promote Persistent Infection
Source: PLoS Pathog. 2010 Jun 10;6(6):e1000944. doi: 10.1371/journal.ppat.1000944 (PMC2883592; doi:10.1371/journal.ppat.1000944)
Supplement: Table S2 — Primers used in this study. (0.02 MB PDF) [file ppat.1000944.s002.pdf]

Table S2. Primers used in this study.

Primer name	Sequence	Comment
P-relA-F-AttB1	CGGGACAAGTTTGTACAAAAAAGCAGGCTTC AACTTCAACCATCATTCG	*relA* ~2kb insertion
P-relA-R-AttB2	GGGGACCACTTTGTACAAGAAAGCTGGGTGG CTTTATCATTGGCTGTCC	
P-CAA-F-attB1	CGGGACAAGTTTGTACAAAAAAGCAGGCTTG GTCAAAACGTTACAAATCATGCCGC	SACOL1230 ~2kb insertion
P-CAA-R-AttB2	GGGGACCACTTTGTACAAGAAAGCTGGGTCG GTACTCATGCCACCGCCG	
ParC-F	TGCATATCGTTGAAGGTTTGA	Confirm *parC* mutation
ParC-R	CATGTTGCCCCAATTCTTTC	
RpoB-F	CCGTCGTTTACGTTCTGTAGG	Confirm *rpoB* mutation
RpoB-R	TTTGGTCCCTCAGGTGTTTC	
